# Effects of recombinant human growth hormone in severe neurosurgical patients: A single center, retrospective study

**DOI:** 10.1371/journal.pone.0317219

**Published:** 2025-01-10

**Authors:** Xixian Liao, Haorun Huang, Binghui Qiu, Jiaping Chen, An Zhang, Haoxin Liang, Chuanping Huang, Fen Mei, Jian Mao, Fan Liu, Ming Jin, Xiaojie Peng, Haidie Ma, Wenjie Ding, Songtao Qi, Yun Bao

**Affiliations:** 1 Department of Neurosurgery, Nanfang Hospital, Southern Medical University, Guangzhou, Guangdong, China; 2 Department of Stomatology, Nanfang Hospital, Southern Medical University, Guangzhou, Guangdong, China; 3 Department of Laboratory for Precision Neurosurgery, Nanfang Hospital, Southern Medical University, Guangzhou, Guangdong, China; 4 Second Clinical Medicine School, Southern Medical University, Guangzhou, Guangdong, China; 5 Department of Gastroenterology, Shenzhen Hospital, Southern Medical University, Shenzhen, Guangdong, China; 6 Department of Critical Care Medicine, Nanfang Hospital, Southern Medical University, Guangzhou, Guangdong, China; 7 First Clinical Medicine School, Southern Medical University, Guangzhou, Guangdong, China; GSVM Medical College, INDIA

## Abstract

**Purpose:**

To explore the effects of recombinant human growth hormone (r-hGH) on inflammatory mediators, immune cells and prognosis in severe neurosurgical patients.

**Methods:**

From August 2020 to June 2021, a total of 236 patients who admitted to the neurosurgical intensive care unit (NSICU) were retrospectively analyzed. The patients were divided into GH group (97 cases) and nGH group (139 cases) according to whether they received r-hGH treatment. Parameters including CD4^+^ T cell counts, inflammatory mediators and prognosis were recorded and assessed.

**Results:**

The results showed that the cure time of pneumonia and intracranial infection in GH group patients was significantly shorter than in the nGH group (24.25 ± 4.89 days and 21.33 ± 1.53 days versus 29.13 ± 7.43 days and 25.17 ± 2.32 days, respectively). However, there was no significant difference in GOS scores between two groups (31.96% ≤ 3 and 68.04% > 3 vs 39.57% ≤ 3 and 60.43% > 3) (*P* = 0.232). Furthermore, the number of CD4^+^ T cells and CD8^+^ T cells in the GH group showed a significant upward trend. Last but not least, significant differences were also observed in IL-6 and IL-10 levels between two groups at days 1, 3, and 7.

**Conclusion:**

The application of r-hGH in severe neurosurgical patients was effective in increasing the number of CD4^+^ T cells, down-regulating inflammatory mediators, shortening the cure time of pneumonia, intracranial infections and urinary tract infections, and improving patients’ prognosis.

## Introduction

Severe neurosurgical patients often experience infections, including respiratory system infections, central nervous system infections, bloodstream infections and urinary tract infections. The incidence of acquired pneumonia in patients with moderate to severe traumatic brain injuries was 30% ~ 61% [1]. A single-center cohort study showed that the incidence of post-admission pneumonia in patients with subarachnoid hemorrhage was 57.1% [2]. With adequate antibiotics use, many patients still have a reduced prognosis or even die due to co-infections [3]. Moreover, the rapid emergence of antibiotic-resistant pathogens as well as the lack of new antimicrobial agents would cause the infection difficult to cure [4, 5]. Therefore, treatments focused on improving patients’ immunity are gaining increasing attention. CD4^+^ T lymphocytes are essential for patients to fight infection [6–8]. Our study found that CD4^+^ T cells and serum IGF-1 levels in severe neurosurgical patients were significantly lower than in normal people [9]. It has been reported that IGF-1 could affect the number of CD4^+^ T cells significantly [10, 11]. Furthermore, CD4^+^ T cell-based therapy can improve patients’ resistance to infections significantly [12]. Therefore, increasing CD4^+^ T cell levels in severe neurosurgical patients may enhance the anti-infection ability of patients and improve the prognosis.

Adult growth hormone deficiency(GHD) is an indication for the use of recombinant human growth hormone [13]. We found that many severe neurosurgical patients also had growth hormone deficiency [14, 15]. It was reported that GH treatment could increase the levels of IGF-1 in the patients [13, 16–19]. Therefore, in this study, we analyzed the changes of the IGF-1, CD4^+^ T cells, CD8^+^ T cells, PCT, CRP, leukocyte and various inflammatory cytokines, and compared the cure time of pneumonia, intracranial infection and urinary tract infection, aiming to investigate the role of GH treatment in improving severe neurosurgical patients’ prognosis.

## Patients and methods

### Study design, inclusion criteria, exclusion criteria and group distribution

We retrospectively studied 236 severe neurosurgical patients in the NSICU of Nanfang Hospital of Southern Medical University, Guangzhou, China from August 2020 to June 2021.

**Fig 1:**
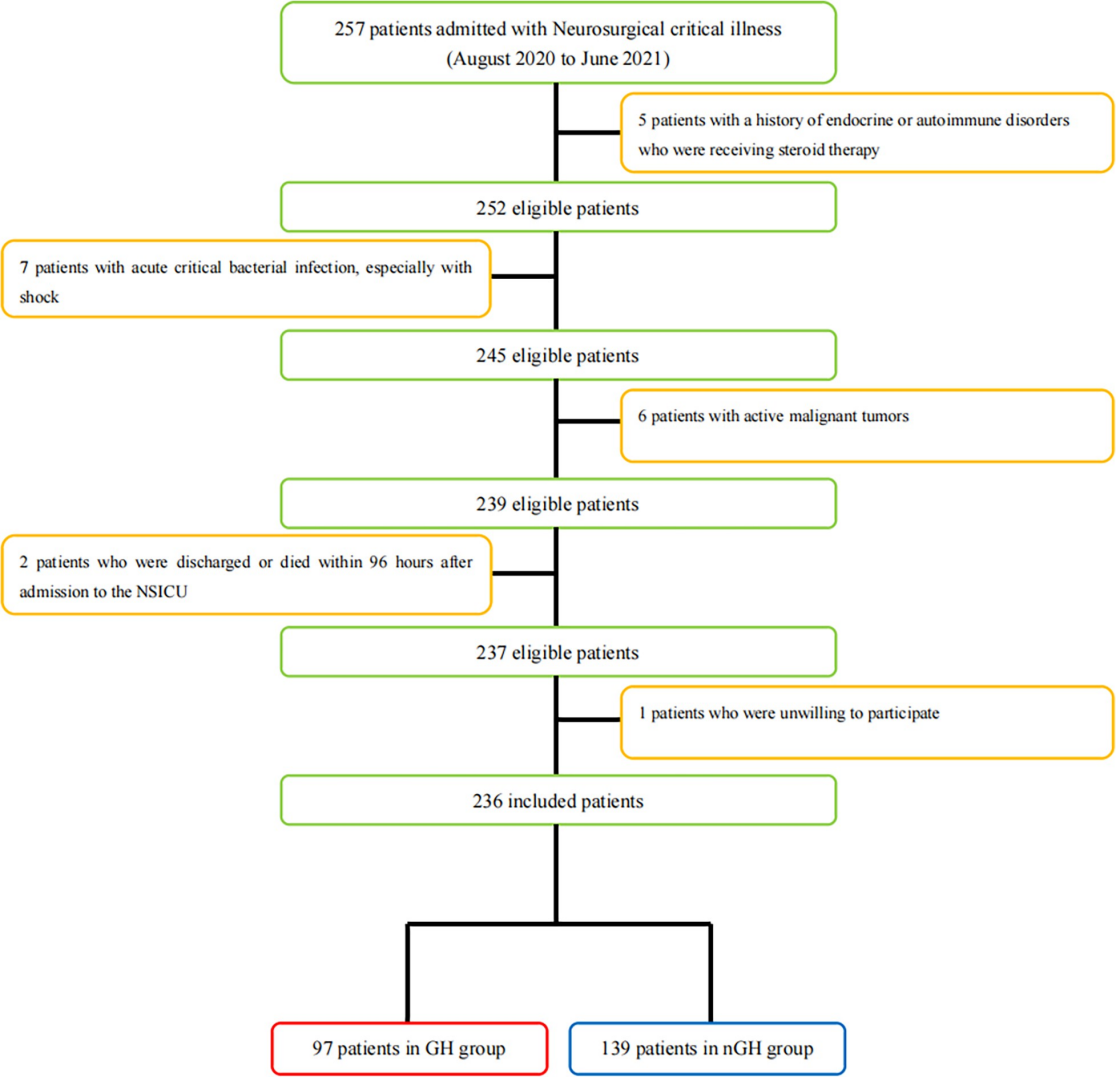
Flow diagram of patient recruitment and inclusion process. NSICU: neurosurgical intensive care unit; GH: growth hormone; nGH: none growth hormone.

Our inclusion criteria were: (1) age≥18 years and ≤80 years; (2) GCS score of ≤ 12 on admission; (3) Stayed in the NSICU for more than 96 hours. Our exclusion criteria were: (1) Patients with a history of endocrine or autoimmune disorders who were receiving steroid therapy; (2) Patients with acute critical bacterial infection, especially with shock; (3)Patients with active malignant tumors; (4) Patients who were discharged or died within 96 hours of admission; (5) Patients who were unwilling to participate (Fig 1).

This study was approved by the Medical Ethics committee of NanFang Hospital of Southern Medical University(NFEC-2023-256). All methods were performed in accordance with the relevant guidelines and regulations of the institution. Oral informed consent was obtained from all participants.

Baseline information including gender, age, admission diagnosis, GCS score at admission, APACHE II score were collected from 236 patients. IGF-1, CD4^+^ T cells, CD8^+^ T cells, PCT, CRP, leukocyte and various inflammatory cytokines in the two groups on days 0, 1, 3 and 7 were recorded and analyzed. At the same time, the incidence and cure time of pulmonary, neurological, bloodstream and urinary tract infections were compared between the two groups. The prognostic indicators were: (1) hospitalization time in the NSICU; (2) NSICU mortality; (3) GOS scores. In particular, the primary outcome was the hospitalization time in the NSICU. Moreover, GOS scores would be evaluated after six months of admission.

### GH treatment regimen

In our study, the levels of the IGF-1 and GH in the patients would be tested on days 0, 1, 3, 7 after admission. If the patients were GH deficiency (defined as peak GH responses to pharmacological stimuli<10ng/ml) and/or the levels of the IGF-1 were lower than 1 standard deviation from the normal value, they would receive the GH therapy within two hours. The r-hGH was provided by Changchun Jinsai Pharmaceutical Co., LTD, and the dose was 0.05 mg/day/kg. The mode of the administration was intramuscular injection and the frequency of administration was once per day. Patients were required to receive the GH therapy for a week unless the levels of the IGF-1 become normal during the treatment.

### The diagnosis of the infection

The diagnosis of pneumonia was based on the combination of typical clinical presentation with confirmatory chest X-ray changes [20, 21]. Patients were diagnosed with intracranial infection when they had fever, central nervous system (CNS) dysfunction (irritability, lethargy, impaired consciousness or coma, and signs of meningeal irritation) and cerebrospinal fluid (CSF) showing lymphocytic pleocytosis and protein levels greater than 40 mg per dL [22]. A positive urine culture was required for the diagnosis of the urinary tract infection [21]. When pathogenic microorganism was isolated from one or more blood cultures and the patients had clinical infection symptoms, including fever (≥38°C) or low temperature (<36°C), shivering, increased (count >10.0×10^9^/L, especially when “nuclear shift to the left”) or decreased (count <3.0×10^9^/L) leukocyte count, skin and mucosa hemorrhage, coma, multiple organ failure, decreased blood pressure, increased C-reactive protein, the patients were diagnosed with a bloodstream infection [23].

### The definition of the cure time

The cure of pneumonia was defined when the typical clinical presentation of patients faded away and the chest X-ray or CT scan showed significant improvement. Intracranial infections were considered cured when the patients had no fever and the CSF test results were normal. Patients were defined as cured of urinary tract infection when the results of the urine culture were normal. Bloodstream infections were defined as cured when the results of the blood cultures were normal and there were no clinical infection symptoms.

### Statistical analysis

Discrete variables were presented as counts and percentages, and compared using chi-square tests. Continuity correction or Fisher’s exact test was used when appropriate. Continuous variables were expressed as mean ± standard deviation and compared by Student t-test or one-way ANOVA. Linear regression was used in the multivariate analysis. Two-sided P<0.05 was considered statistically significant. All statistical analyses were performed using IBM SPSS statistics software (version 18.0).

## Results

A total of 236 patients were included in the study, including 97 cases in GH group and 139 cases in nGH group. There were 151 males and 85 females with an average age of 50.15 ± 14.51 years old. There was no significant difference in age, gender, GCS scores, APACHE II score and the disease between the two groups (*P* = 0.356, 0.263, 0.514, 0.344, 0.953) (Table 1). On day 0, there was a positive correlation between IGF-1 and CD4^+^ T cell levels in patients’ blood, and the correlation coefficient was 0.727 (*P* < 0.001) (S1 Fig).

**Table 1:** Patient characteristics.

	GH	n-GH	Total	P Value
Sex (NO.)				0.263
Male	58 **(**59.79**)**	93 **(**66.91**)**	151 **(**63.98**)**	
Female	39 **(**40.21**)**	46 **(**33.09**)**	85 **(**36.02**)**	
Age, mean (range)	49.10±14.44/	50.88±14.56/	50.15±14.51/	0.356
	**(**19–76**)**	**(**19–80**)**	**(**19–80**)**	
Admission				0.514
GCS (NO.)				
9~12	17 **(**17.53**)**	20 **(**14.39**)**	37 **(**15.68**)**	
3~8	80 **(**82.47**)**	119 **(**85.61**)**	199 **(**84.32**)**	
APACHE II				0.344
(NO.)				
≤15	62 **(**63.92**)**	97 **(**69.78**)**	159 **(**67.37**)**	
>15	35 **(**36.08**)**	42 **(**30.22**)**	77 **(**32.63**)**	
Disease (NO.)				0.953
Subarachnoid hemorrhage,	17 **(**17.53**)**	23 **(**16.55**)**	40 **(**16.95**)**	
n (%)				
Cerebral hemorrhage,	31 **(**31.96**)**	52 **(**37.41**)**	83 **(**35.17**)**	
n (%)				
Brain stem	11 **(**11.34**)**	15 **(**10.79**)**	26 **(**11.02**)**	
hemorrhage,				
n (%)				
Arterial aneurysm,	7 **(**7.22**)**	11 **(**7.91**)**	18 **(**7.63**)**	
n (%)				
cerebral infarction,	5 **(**5.15**)**	4 **(**2.88**)**	9 **(**3.81**)**	
n (%)				
Traumatic brain injury,	25 **(**25.77**)**	32 **(**23.02**)**	57 **(**24.15**)**	
n (%)				
Others, n (%)	1 **(**1.03**)**	2 **(**1.44**)**	3 **(**1.27**)**	

Analysis of variance and Chi-square test were used to compare continuous and categorical variables, respectively. Admission GCS, Admission Glasgow coma score; APACHE II, Acute Physiology and Chronic Health Evaluation II.

**Fig 2:**
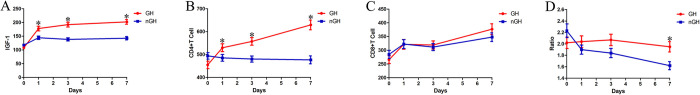
The comparison of the IGF-1, CD4^+^ T cell, CD8^+^ T cell and CD4^+^ T cell/CD8^+^ T cell between the GH group and the nGH group. A. The comparison of IGF-1; B. The comparison of CD4^+^ T cell; C. The comparison of CD8^+^ T cell; D. The comparison of CD4^+^ T cell/CD8^+^ T cell. *: *P* < 0.05.

The results showed that the plasma IGF-1 levels were significantly elevated after 7 days of GH treatment (*P* < 0.001) (Fig 2A). Furthermore, no significant differences existed in CD4^+^ T cells, CD8^+^ T cells and CD4^+^ T cell/CD8^+^ T cell between the two groups before r-hGH treatment (P values were 0.074; 0.335; 0.189, respectively). As shown in Fig 2, from day 0 to day 7, there was an increase in CD4^+^ T cells and CD8^+^ T cells numbers in the GH group as well as an increase in CD8^+^ T cells numbers and a slight decrease in CD4^+^ T cells numbers in the nGH group. Moreover, a decrease in CD4^+^ T cell/CD8^+^ T cell ratio in the nGH group was also observed from day 0 to 7 while no change in the GH group was observed (*P* = 0.393). There was also a significant increase in CD4^+^ T cells on days 1, 3, and 7 compared to the nGH group (P = 0.049, P<0.001, P<0.001). No significant difference between the two groups in CD8^+^ T cells numbers on day 1, 3, or 7 was observed (P values were 0.963, 0.708, 0.245). In addition, there was a significant increase in CD4^+^ T cell/CD8^+^ T cell ratio in the GH group compared to the nGH group at day 7 (*P* = 0.003), and no significant differences were found at day 1 or 3 (P values were 0.290, 0.071).

As shown in Table 2, the incidence of pneumonia, intracranial infections, bloodstream infections and urinary tract infections did not differ between GH group and nGH group (73.20%, 3.09%, 2.06%, 6.19% vs 74.82%, 4.32%, 2.88%, 7.19%, *P* = 0.779, 0.629, 0.695, 0.762, respectively). The cure time of pneumonia and intracranial infections in the GH group was significantly shorter than in the nGH group (24.25 ± 4.89 days and 21.33 ± 1.53 days vs 29.13 ± 7.43 days and 25.17 ± 2.32 days, respectively). No significant difference was observed in the cure time of bloodstream infections and urinary tract infections between the two groups (8.50 ± 0.71 days and 13.83 ± 1.17 days vs 9.25 ± 1.26 days and 15.10 ± 2.28 days, respectively), but the hospitalization time in NSICU in GH group was significantly shorter than in nGH group (20.41 ± 7.54 days vs 26.14±7.89 days, *P* < 0.001). However, there was no difference in GOS scores between the two groups (31.96% ≤ 3 and 68.04%>3 vs 39.57% ≤ 3 and 60.43%>3, *P* = 0.232). Furthermore, there was no difference in NSICU mortality between the two groups (*P* = 0.744).

**Table 2:** Data on concurrent infections were obtained for all enrolled patients.

	GH	n-GH	Total	P Value
Pneumonia (NO.)				0.779
Yes, n (%)	71 **(**73.20**)**	104 **(**74.82**)**	175 **(**74.15**)**	
No, n (%)	26 **(**26.80**)**	35 **(**25.18**)**	61 **(**25.85**)**	
The Treatment Time, days±mean	24.25±4.89	29.13±7.43	27.15±6.93	<0.001*
Intracranial Infection (NO.)				0.629
Yes, n (%)	3 **(**3.09**)**	6 **(**4.32**)**	9 **(**3.81**)**	
No, n (%)	94 **(**96.91**)**	133 **(**95.68**)**	227 **(**96.19**)**	
The Treatment Time, days±mean	21.33±1.53	25.17±2.32	23.89±2.76	0.038*
Blood Infection (NO.)				0.695
Yes, n (%)	2 **(**2.06**)**	4 **(**2.88**)**	6 **(**2.54**)**	
No, n (%)	95 **(**97.94**)**	135 **(**97.12**)**	230 **(**97.46**)**	
The Treatment Time, days±mean	8.50±0.71	9.25±1.26	9.00±1.10	0.492
Urinary Tract Infection (NO.)				0.762
Yes, n (%)	6 **(**6.19**)**	10 **(**7.19**)**	16 **(**6.78**)**	
No, n (%)	91 **(**93.81**)**	129 **(**92.81**)**	220 **(**93.22**)**	
The Treatment Time, days±mean	13.83±1.17	15.10±2.28	14.63±2.00	0.231
NSICU Length of Stay, days±mean	20.41±7.54	26.14±7.89	23.79±8.23	<0.001*
NSICU mortality	4 **(**4.02**)**	7 **(**5.04**)**	11 **(**4.66**)**	0.744
GOS scores (NO.)				0.232
≤3	31 **(**31.96**)**	55 **(**39.57**)**	86 **(**36.44**)**	
>3	66 **(**68.04**)**	84 **(**60.43**)**	150 **(**63.56**)**	

Analysis of variance and Chi-square test were used to compare continuous and categorical variables, respectively. NSICU, Neurosurgery intensive care unit; GOS, Glasgow Outcome Scale.

*P<0.05

**Fig 3:**
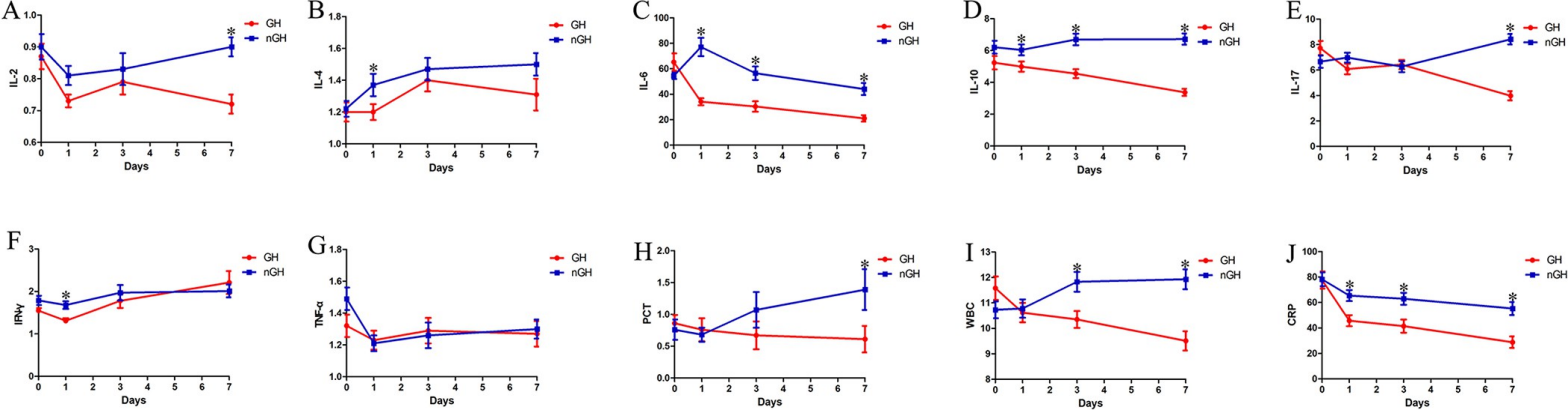
The comparison of the IL-2, IL-4, IL-6, IL-10, IL-17, IFN-γ, TNF-α, PCT, WBC and CRP between the GH group and the nGH group. A. The comparison of IL-2; B. The comparison of IL-4; C. The comparison of IL-6; D. The comparison of IL-10; E. The comparison of IL-17; F. The comparison of IFN-γ; G. The comparison of TNF-α; H. The comparison of PCT; I: The comparison of WBC; J. The comparison of CRP. *: *P* < 0.05.

In order to evaluate the effects of r-hGH therapy on serum cytokine levels in patients in NSICU, blood samples were collected on 0, 1, 3 and 7 days after the treatment, in which the concentrations of IL-2, IL-4, IL-6, IL-10, IL-17, TNF-α and INF-γ were analyzed. Before the treatment, there was no significant differences in the serum levels of IL-2, IL-4, IL-6, IL-10, IL-17, IFN-γ and TNF-α between the two groups. As shown in Fig 3, during the treatment of r-hGH, the levels of IL-2, IFN-γ, IL-6, IL-10 and IL-17 showed changes (*P* = 0.011, 0.021, < 0.001, < 0.001, < 0.001), while IL-4 and TNF-α levels showed none (*P* = 0.242, 0.604). In addition, IL-6 and IL-10 levels showed a significant difference between two groups on days 1, 3, and 7 (Fig 3C and 3D), however, IFN-γ and TNF-α levels indicated no significant differences between the two groups on days 3 and 7 (Fig 3F and 3G).

To further evaluate the influence of r-hGH therapy on other serum inflammatory biomarkers in patients, the concentrations of CRP, WBC and PCT in blood samples were collected on the 0, 1, 3 and 7 days after treatment were analyzed. Before the treatment, no significant differences were observed in CRP, WBC and PCT between the two groups. At day 7 of the treatment, these three biomarkers showed significant changes (Fig 3H–3J).

Furthermore, we found that there weren’t any differences between the male and female patients for CD4^+^ T cells, CD8^+^ T cells, IGF-1, cytokine levels in response to the GH therapy(S2 and S3 Figs). Moreover, we also found that there weren’t any age effects on the response to GH therapy (S4 and S5 Figs).

Last but not least, we performed a multivariate analysis of the factors that may influence the NSICU time of the patients. The results showed that the gender, the admission GCS, APACHE II and the GH treatment were the significant influential factors to the NSICU time of the patients **(***P* = 0.012, 0.002, 0.033, <0.001**)** (Table 3). Moreover, as shown in Table 3, the age and the disease were not the influential factors to the NSICU time of the patients (*P* = 0.928, 0.965).

**Table 3:** Multivariate analysis of factors influencing the NSICU time in two groups.

Factors	βValue	P Value	βValue (95%CI)
GH Treatment	-5.770	<0.001*	**(**-7.727,-3.813**)**
Sex	2.786	0.012*	**(**0.617,4.955**)**
Age	0.067	0.928	**(**-1.393,1.526**)**
Admission GCS	-0.636	0.002*	**(**-1.033,-0.239**)**
APACHE II	0.254	0.033*	**(**0.021,0.486**)**
Disease	0.011	0.965	**(**-0.502,0.525**)**

Linear regression was used in the multivariate analysis.*P<0.05

## Discussion

The treatment of patients with severe neurosurgical illness has been challenging. Patients with severe neurosurgical illness are often have psychiatric disorders and decreased pituitary-hypothalamic function [24–28], which further leads to a decrease in patients’ immunity. As a result, patients have a high rate of infection and are difficult to treat. In the earlier part of this study, the IGF-1 levels in severe neurosurgical patients were found to be significantly lower than in normal people. At the same time, previous literature reported that IGF-1 can significantly affect the immune function of non-neurologically severe patients [29–31]. However, there are no reports on the immune function of patients with severe neurological illness. In order to investigate the possibility of improved immune function after elevated IGF-1 in severe neurological patients, this study used retrospective analysis to compare the cellular immune function, infectious indicators and prognosis in patients with or without r-hGH treatment.

Our study found that there was a positive correlation between serum IGF-1 and the number of CD4^+^ T cells in neurosurgical critically ill patients.

In our study, severe neurological patients had decreased CD4^+^ T cell counts and a high incidence of pulmonary infections, while patients treated with r-hGH had increased CD4^+^ T and CD8^+^ T cell counts. CD4^+^ T cells can assist CD8^+^ T cells to produce effective immunity against pathogenic microorganisms such as viruses and bacteria, so as to kill pathogenic microorganisms and protect the body [32–34]. In addition, human growth hormone (hGH) aggregates induced dendritic cells (DCs) maturation with notably an increase in CXCL10 production, which played an important role in the CD4^+^ T cells dependent adaptive immune response [35]. Our results showed that r-hGH treatment was effective in shortening the cure time of pneumonia and intracranial infections in severe neurosurgical patients and increasing CD4^+^ T cell counts.

It was previously reported that GH may increase the activity of immune cells and promote the expression of inflammatory cytokines [36–38], and affect the differentiation of the CD4+ T cells through *Growth hormone* secretagogue receptor 1a (GHSR1a) [39]. Moreover, an animal study showed that the combination of GH and IGF-1 promoted thymocyte transport, which also corroborated this hormone’s ability to promote thymocyte motility [40]. However, r-hGH treatment decreased the inflammatory cytokines such as IL-6. Besides, r-hGH treatment also reduced the levels of infection indexes (PCT, CRP, and WBC) of patients. Accordingly we believed that the decrease in IL-6 was caused by a decrease in the level of infection.

In terms of other endocrine axes, there was no significant difference in the incidence of hypothyroidism in the thyroxine and cortisol axes between the GH group and nGH group, indicating that r-hGH did not antagonize other endocrine axis hormones, resulting in their hypofunction. In previous studies, it has been reported that r-hGH can improve the function of various cells.

The main limitation of this study was its retrospective nature. We had to rely on the correct filling of medical records and the clinical assessment of the attending physician at the time. In addition, this was a single-center study which may limit the generalizability of the final results. Therefore, to further verify our conclusion, we would do the randomized controlled trial which has a large sample size. Furthermore, there was an important limitation of the study that we may not have taken full confounding factors into account. So we would consider as many confounding factors as possible in the randomized controlled trial.

## Conclusion

In conclusion, r-hGH increased the number of the CD4^+^ T cells in severe neurosurgical patients, down-regulated inflammatory mediators, shortened the cure time of pneumonia and intracranial infection, and improved the prognosis of patients. Therefore, if the level of the IGF-1 in severe neurosurgical patients is low, they should receive the GH therapy appropriately.

## Supporting information

S1 Fig The correlation between IGF-1 and CD4^+^ T cells in patients’ blood on day 0. R = 0.7265, *P* < 0.0001.
(TIF)

S2 Fig The comparison of the IGF-1, CD4^+^ T cell, CD8^+^ T cell and CD4^+^ T cell/CD8^+^ T cell between the male and the female patients in the GH group. A. The comparison of IGF-1; B. The comparison of CD4^+^ T cell; C. The comparison of CD8^+^ T cell; D. The comparison of CD4^+^ T cell/CD8^+^ T cell. *: *P* < 0.05.
(TIF)

S3 Fig The comparison of the IL-2, IL-4, IL-6, IL-10, IL-17, IFN-γ, TNF-α, PCT, WBC and CRP between the male and the female patients in the GH group. A. The comparison of IL-2; B. The comparison of IL-4; C. The comparison of IL-6; D. The comparison of IL-10; E. The comparison of IL-17; F. The comparison of IFN-γ; G. The comparison of TNF-α; H. The comparison of PCT; I: The comparison of WBC; J. The comparison of CRP. *: *P* < 0.05.
(TIF)

S4 Fig The comparison of the IGF-1, CD4^+^ T cell, CD8^+^ T cell and CD4^+^ T cell/CD8^+^ T cell between different age groups in the GH group. A. The comparison of IGF-1; B. The comparison of CD4^+^ T cell; C. The comparison of CD8^+^ T cell; D. The comparison of CD4^+^ T cell/CD8^+^ T cell. *: *P* < 0.05.
(TIF)

S5 Fig The comparison of the IL-2, IL-4, IL-6, IL-10, IL-17, IFN-γ, TNF-α, PCT, WBC and CRP between different age groups in the GH group. A. The comparison of IL-2; B. The comparison of IL-4; C. The comparison of IL-6; D. The comparison of IL-10; E. The comparison of IL-17; F. The comparison of IFN-γ; G. The comparison of TNF-α; H. The comparison of PCT; I: The comparison of WBC; J. The comparison of CRP. *: *P* < 0.05.
(TIF)

S1 File Dataset. (XLSX)
