# Supplementary figures and images for: Effects of recombinant human growth hormone in severe neurosurgical patients: A single center, retrospective study
Source: PLoS One. 2025 Jan 10;20(1):e0317219. doi: 10.1371/journal.pone.0317219 (PMC11723630; doi:10.1371/journal.pone.0317219)

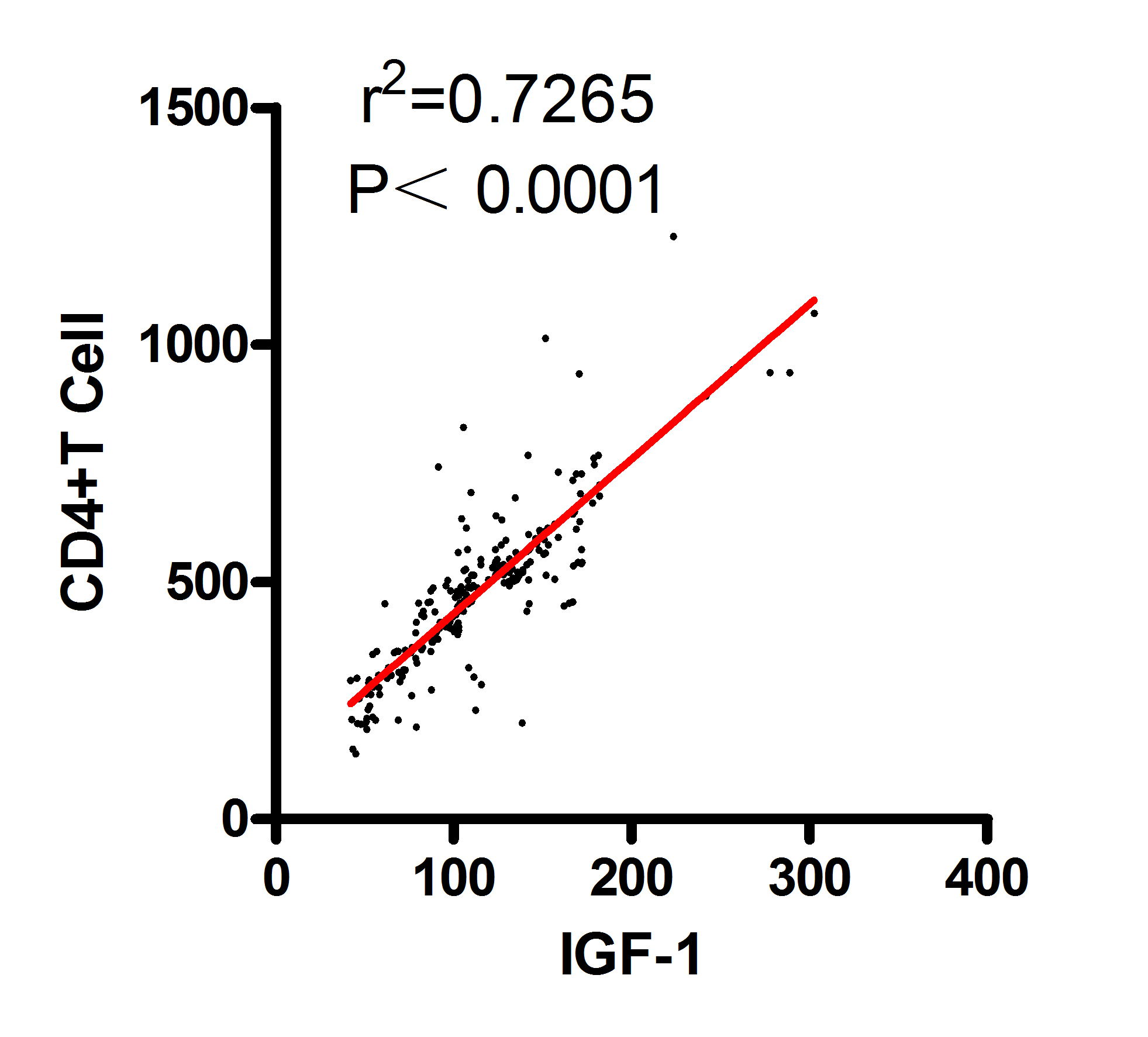

Supplement: S1 Fig — R = 0.7265, P < 0.0001. (TIF) [file pone.0317219.s001.tif]

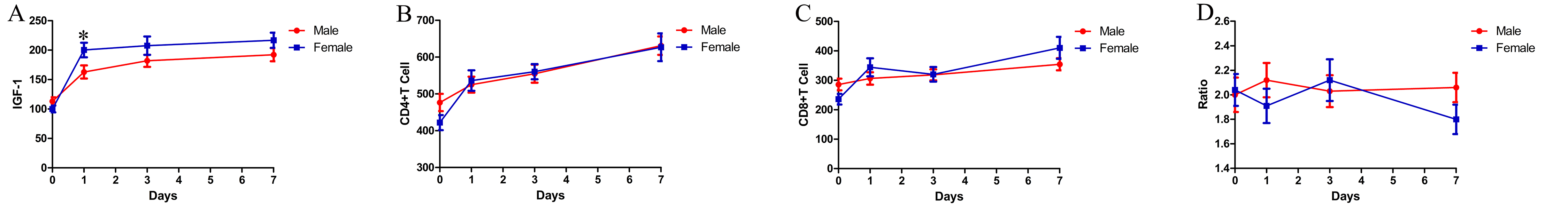

Supplement: S2 Fig — A. The comparison of IGF-1; B. The comparison of CD4+ T cell; C. The comparison of CD8+ T cell; D. The comparison of CD4+ T cell/CD8+ T cell. *: P < 0.05. (TIF) [file pone.0317219.s002.tif]

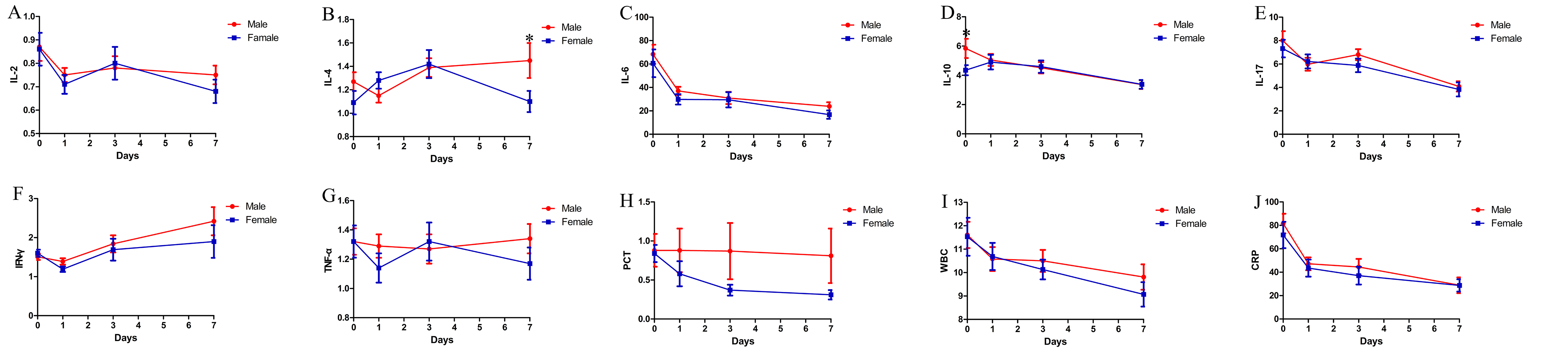

Supplement: S3 Fig — A. The comparison of IL-2; B. The comparison of IL-4; C. The comparison of IL-6; D. The comparison of IL-10; E. The comparison of IL-17; F. The comparison of IFN-γ; G. The comparison of TNF-α; H. The comparison of PCT; I: The comparison of WBC; J. The comparison of CRP. *: P < 0.05. (TIF) [file pone.0317219.s003.tif]

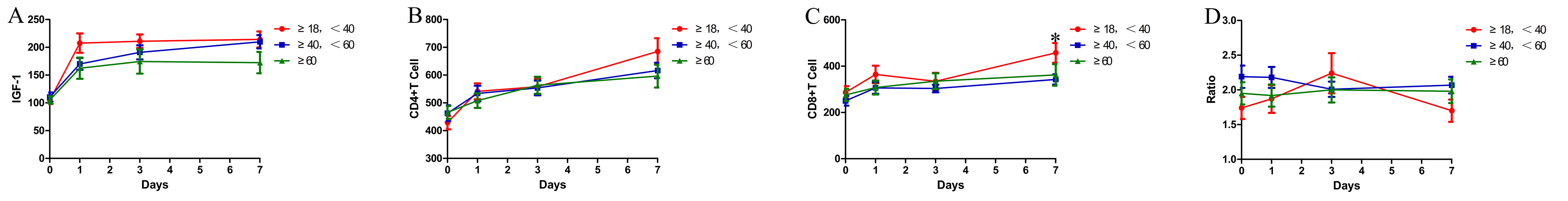

Supplement: S4 Fig — A. The comparison of IGF-1; B. The comparison of CD4+ T cell; C. The comparison of CD8+ T cell; D. The comparison of CD4+ T cell/CD8+ T cell. *: P < 0.05. (TIF) [file pone.0317219.s004.tif]

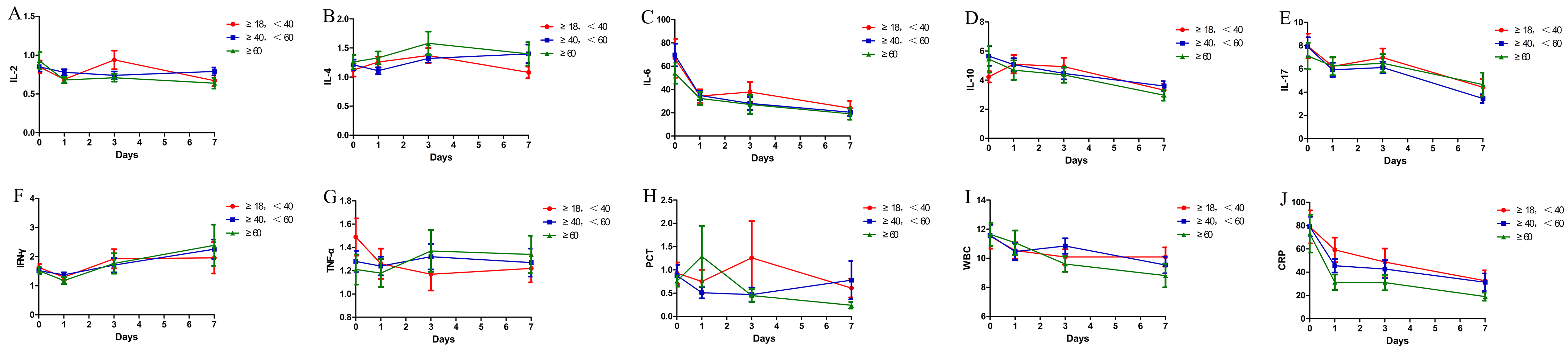

Supplement: S5 Fig — A. The comparison of IL-2; B. The comparison of IL-4; C. The comparison of IL-6; D. The comparison of IL-10; E. The comparison of IL-17; F. The comparison of IFN-γ; G. The comparison of TNF-α; H. The comparison of PCT; I: The comparison of WBC; J. The comparison of CRP. *: P < 0.05. (TIF) [file pone.0317219.s005.tif]
